# The need for more medical schools in medically underserved regions in Africa

**DOI:** 10.1016/j.amsu.2022.104967

**Published:** 2022-11-17

**Authors:** Farida Oluwabukola Brimmo, Abdulhammed Opeyemi Babatunde, Naomi Nmesoma Ezefuna, Mary Sathela Kanu, Patrick Biziyaremye

**Affiliations:** Standing Committee on Medical Education and Research, Federation of African Medical Students' Associations (FAMSA), Ibadan, Nigeria; Medicine and Surgery, Faculty of Clinical Sciences, College of Medicine, University of Ibadan, Ibadan, Nigeria; Standing Committee on Medical Education and Research, Federation of African Medical Students' Associations (FAMSA), Ibadan, Nigeria; Medicine and Surgery, Faculty of Clinical Sciences, College of Medicine, University of Ibadan, Ibadan, Nigeria; Standing Committee on Medical Education and Research, Federation of African Medical Students' Associations (FAMSA), Ibadan, Nigeria; University of Nigeria, Nsukka, Nigeria; Standing Committee on Medical Education and Research, Federation of African Medical Students' Associations (FAMSA), Ibadan, Nigeria; College of Medicine and Allied Health Sciences, University of Sierra Leone, Freetown, Sierra Leone; Standing Committee on Medical Education and Research, Federation of African Medical Students' Associations (FAMSA), Ibadan, Nigeria; Medicine and Surgery, University of Rwanda, Kigali, Rwanda

**Keywords:** Medical school, Africa, Physician shortage, Healthcare, Medical education

## Abstract

Africa struggles with the double burden of disease, bearing the highest disease burden in the world and also having the most severe health workforce shortage. Only four countries on the continent meet the WHO-recommended density of 4.45 health workers per 1000 people. This physician shortage has been attributed to a variety of factors including shortfalls in medical education and medical schools' capacities. This commentary aims to reveal the gap and ‘underrated’ problem of inadequate medical schools and poor utilization of existing ones. Recommended solutions calling for the need for urgent improvement in medical education in Africa are highlighted in the paper.

## Commentary

Africa faces a critical shortage of physicians, which has significantly affected the continent's suboptimal quality of health care delivery [1]. Although Africa struggles with the double burden of disease, bearing the highest disease burden of the world, it has the most severe health workforce shortage [2]. The continent also struggles with several other poor health indicators qualifying the region to be categorized as medically underserved. Medically underserved regions have been defined as an area with shortage of health care services indicated by high infant mortality rate, the proportion of the population living in poverty and the percentage of the elderly population, as well as a low health provider to population ratio [3]. There has been a persistent physician shortage in African nations, with about 0.31 million doctors across the continent [4]. The majority of countries in the continent have fewer than the WHO-recommended density of 4.45 health workers per 1000 people, which is required to provide basic services with only four nations: Seychelles, Namibia, Mauritius, and South Africa having an adequate number of health care workers [4,5]. Somalia, which has only 0.023 physicians for every 1000 people, ranks first on this list of nations with the fewest physicians per person worldwide, followed by other twenty African countries with a physician-to-population ratio ranging from 0.036 to 0.093 per 1000. This stands in stark contrast from western countries like the United Kingdom and France, where there are 5.8 doctors per 1000 people, and 6.5 doctors per 1000 people, respectively [6]. This physician shortage has been attributed to a variety of factors such as the widespread foreign migration as European countries offer better wages, working and training conditions, career change, rapid population expansion, and shortfalls in medical education [7].

The deficiencies in medical education in Africa significantly impact the physician shortage as we are simply not training sufficient physicians to cater to the population [8]. Medical education in Africa is facing multiple challenges such as underfunding, shortage of faculty staff, poor infrastructure, and shortage of medical schools [9]. The continent is home to around 11% of the world's medical schools, while the United States of America alone has about 8% of the world's medical schools. In contrast to Asia and Europe, Africa also has an unequal distribution of ites few medical schools. Despite having the fewest schools in the region, Southern Africa's schools per million inhabitants at 0.21 is higher than that of Central Africa and Western Africa (Table 1). Further disparities in distribution exist within these regions. For example, only one medical school can be found in each of the seven West African nations (Guinea, Liberia, Mali, Niger, Sierra Leone, Burkina Faso, and Togo), whereas 45 medical schools are located in Nigeria alone [10,11]. A total of 18 countries in the continent have just one medical school. However, in two of these countries—Seychelles and Namibia—the number of health workers is above the WHO-recommended density owing to the small population. This may imply that they need only one medical school to produce sufficient physicians to cater for the nation. This is not the case for other countries with one or more medical schools, such as Madagascar, Malawi, Togo, Benin, South Sudan, Chad, Central African Republic, and Niger, where the ratio of medical schools per million inhabitants ranges between 0.05 and 0.2. Less than 0.5 health workers per 1000 people are present in these countries [4,10,11].

**Table 1:** Distribution of active medical schools across the five African Regions.

African Region	Number of Medical Schools	Estimated Population	Medical Schools per million population
Eastern Africa	113	461 million	0.26
Northern Africa	111	256 million	0.43
Western Africa	70	419 million	0.17
Central Africa	22	190 million	0.12
Southern Africa	14	68 million	0.21

A table showing the distribution of active medical schools across the five African Regions and the ratio of population to medical schools [10,11].

Addressing the problem of limited and uneven distribution of medical schools in Africa is critical to improving physician supply. The establishment of medical schools in these areas is crucial, but it can be challenging and expensive. To reduce the cost, the existing medical schools should increase their capacities by the creation of annex campuses [12]. To accommodate for the increase in medical students, the government can invest in building more teaching hospitals and upgrading private and non-governmental organizations owned health facilities into teaching hospitals. For instance, the best performing district hospitals in Burkina Faso could be upgraded into teaching hospitals awarding medical degrees. Government can increase these hospitals' capacities and convert them into teaching hospitals where students from branch campuses can receive training. In addition to the quantity and capacity of medical schools, it is essential to consider the quality of education. Therefore, collaboration with foreign medical schools and medical research groups through exchange programs should be considered as well. Such collaboration could be advantageous for both parties as students experience the practices of medicine in different cultures. Furthermore, this allows medical students in Africa access to the latest knowledge and best medicine practices.

Another crucial step is to review the admission criteria into medical schools. The majority of schools in Africa only select students based on their cognitive skills in science subjects [13]. As medicine is as much an art as it is a science, a high degree of cognitive ability in sciences and humanities combined with considerable components of psychosocial competency is required for successful completion of medical school and subsequent transformation into a compassionate healthcare provider. These African institutions should consider a more holistic approach and prioritize non-cognitive factors such as conducting personality tests (like Myers-Briggs Personality Test), interpersonal skills, and leadership potential to choose candidates who can successfully develop into competent well-rounded physicians. Besides, integrating medical students in clinical years into the community through more structured and longer community medicine rotations can reduce the workload on the limited health workforce in local communities while also providing more space in teaching hospitals to accommodate more students. This will sharpen their skills in diagnosing common medical conditions rather than spending more time observing sophisticated procedures in teaching hospitals. Finally, the utilization of cutting-edge technology in medical education, including telemedicine, virtual reality, robotic mannequins, and artificial intelligence, will raise the quality of education and open up opportunities for distance or remote learning.

In conclusion, it is worrisome that despite the shortage of medical doctors in Africa, the continent also suffers from inadequate or underutilized medical schools. This is a call to action for the government, private organizations, non-governmental organizations, and major stakeholders to improve medical education in African nations to produce more qualified doctors in an effort to uplift Africa's healthcare system and reduce health inequalities.

## Declaration of competing interest

The authors have no competing interest to declare.

## Ethical approval

Not applicable.

## Sources of funding

No funding was received.

## Author contribution

AOB conceptualized the manuscript. FOB, NME, MSK and PB drafted the manuscript. AOB and FOB reviewed the manuscript. All authors approved the manuscript for submission.

## Registration of research studies

Name of the registry:

Unique Identifying number or registration ID:

Hyperlink to your specific registration (must be publicly accessible and will be checked):

## Guarantor

Not applicable.

## Consent

Not applicable.

## Declaration of competing interest

Authors declare no conflict of interest.
